# Affective homophily as the dominant organizing principle in online conflict discourse networks

**DOI:** 10.1038/s41598-025-19605-3

**Published:** 2025-10-10

**Authors:** Zhexi Gu, Runping Zhu, Fushi Bian

**Affiliations:** 1 School of Information and Library Science, University of North Carolina at Chapel Hill, Chapel Hill, NC USA; 2 School of Humanities and Social Sciences, Beijing Normal-Hong Kong Baptist University, Zhuhai, China; 3 Department of Digital Media and Technology, Lanzhou University, Lanzhou, China

**Keywords:** Computational social science, Graph attention network, Affective homophily, Network brokerage, Polarization, Mathematics and computing, Psychology, Psychology, Science, technology and society

## Abstract

This study develops a diagnostic framework using a Graph Attention Network (GATv2) to uncover the organizing principles of online conflict networks. By modeling link prediction and rigorously comparing GATv2 against multiple baselines, we validate its superior performance and interpretability. Our analysis delivers a “computational verdict”: the model learns to overwhelmingly prioritize affective homophily while systematically discounting users’ structural prestige. Probing the learned embeddings reveals that emotion is the dominant dimension of this homophily, a phenomenon we term “emotional fortresses.” Critically, by analyzing the model’s prediction failures, we identify a novel class of “emergent brokers” who bridge emotional divides through low centrality and affective neutrality. Finally, applying the framework to non-conflict domains empirically establishes the theory’s boundary conditions. This research provides a new theoretical lens and methodological tool for understanding network dynamics in polarized environments.

## Introduction

In 2025, multiple international conflicts—including tensions between India-Pakistan, Iran-Israel, and Russia-Ukraine—intensified simultaneously, drawing unprecedented global attention. However, unlike previous geopolitical crises, a critical battlefield for these conflicts emerged in the digital public sphere^1^. In China, social media platforms such as Sina Weibo rapidly became the main arenas for the public to construct meaning, form opinions, and express stances regarding these international events^2^. The posts, retweets, and comments interwove into a complex web of discourse, reflecting and influencing public perception and emotion towards this international conflict in real-time. This phenomenon illuminates a broader scholarly challenge, during moments of high uncertainty such as international crises, digital public spheres evolve into complex information ecosystems where information, emotion, and influence interact in ways we do not yet fully comprehend^3^. The dynamics operating on non-Western platforms like Weibo may differ significantly from research conclusions based on Western platforms such as Twitter, highlighting the need for platform-specific and culturally contextual investigations of crisis discourse^2,4^. Understanding how crisis discourse unfolds in digital public spheres has become increasingly critical for several reasons. First, social media platforms now serve as primary sources of information during rapidly evolving international events, making their influence mechanisms central to public opinion formation and political behavior^5,6^. Second, the speed and scale at which information propagates through these networks can amplify both accurate reporting and misinformation, potentially affecting real-world diplomatic and military responses^7^. Third, the emotional dynamics of online crisis discourse may contribute to political polarization and inter-group conflict, with implications extending far beyond the digital realm^8^.

The theoretical significance of this inquiry lies in its potential to advance our understanding of three fundamental debates in computational social science: the distinction between influence and homophily in information diffusion^9^, the role of emotion in network dynamics^10^, and the functional differentiation of structural brokers in polarized environments^11,12^. These debates, while individually well-established, have rarely been examined within a unified analytical framework during high-stakes crisis situations.

This study develops an integrative analytical framework using Graph Neural Networks (GNNs) to examine network formation through the task of link prediction. Unlike traditional methods that analyze structure, semantics, and emotion as separate variables, our framework uses end-to-end learning to generate user representations that capture the complex interplay between these dimensions. The core methodological innovation is a shift from disaggregated statistical analysis to integrated representation learning. This allows us to observe how different forces interact within a shared latent space to shape network dynamics, moving beyond classic binaries—such as influence versus homophily—to understand how these factors co-evolve and mutually constitute the structure of online discourse. Our research offers both methodological and theoretical contributions. Methodologically, we introduce a novel application of GNNs to crisis discourse, demonstrating their capacity to capture complex interactions missed by traditional statistics. Theoretically, we advance the understanding of how structural, semantic, and emotional factors jointly shape network dynamics. Our specific contributions are threefold: we (1) empirically distinguish between influence and homophily effects in crisis networks; (2) map the emotional polarization landscape and its structural embedding; and (3) identify an emergent typology of network brokers that challenges the traditional mediator-agitator dichotomy.

## Literature review

The analysis of crisis discourse in digital public spheres intersects with three fundamental debates that have shaped computational social science over the past two decades. These debates—concerning the distinction between influence and homophily, the role of emotion in information diffusion, and the functional roles of structural brokers—have traditionally been examined in isolation. This literature review establishes the theoretical foundation for an integrative framework that addresses these debates simultaneously within the context of international crisis discourse.

### Influence versus homophily: the diffusion dilemma

Distinguishing influence from homophily—the tendency for similar individuals to connect—is a classic challenge in network science, with seminal work revealing that a large portion of apparent social contagion is attributable to homophily, necessitating more advanced methods to disentangle the two. Homophily, defined as the tendency for similar individuals to form connections, can create behavioral correlations across network ties that may be mistakenly attributed to peer influence or social contagion^13^. This distinction is crucial because it addresses fundamentally different mechanisms of social behavior: influence suggests that individuals change their attitudes or behaviors in response to their network connections, while homophily suggests that individuals with similar predispositions are more likely to connect in the first place^14^. Early network studies often conflated these mechanisms, leading to what Aral et al.^15^ termed “contagion confusion.” Their seminal work demonstrated that up to 50% of apparent peer effects in product adoption could be attributed to homophily rather than influence, fundamentally challenging assumptions about social contagion in networks. Subsequent methodological advances have focused on temporal analysis^16^, instrumental variables^17^, and randomized field experiments^18^ to disentangle these effects.

### The emotion paradox in information diffusion

The role of emotion in information diffusion has generated contradictory findings that reveal what we term the “emotion paradox.” On one hand, numerous studies have identified negative emotions, particularly anger and moral outrage, as powerful drivers of viral content^19,20^. The “viral fuel” hypothesis suggests that emotionally charged content receives more engagement and achieves broader reach than neutral content^21^. However, other research has challenged this straightforward relationship. Some studies find that once network structure and user characteristics are controlled for, the direct effect of emotional content diminishes^22^. Additionally, research on emotional contagion suggests that while emotions spread through networks, the mechanisms and outcomes differ significantly from those of information diffusion^23^. Political communication research has provided mixed evidence regarding emotion’s role in political information diffusion. Tucker et al.^24^ found that angry tweets about political topics receive more retweets than neutral ones, while Jost et al.^25^ demonstrated that fear-based political messages are more likely to be shared among conservatives than liberals. However, these effects appear to be moderated by political identity, network position, and the specific type of emotional appeal^26^. The complexity deepens when considering cross-cultural differences in emotional expression and response. Research on Chinese social media platforms has revealed different patterns of emotional expression compared to Western platforms, with implications for how emotional content diffuses^27^. Studies of Weibo during various crises have shown that while negative emotions initially drive rapid diffusion, positive emotions may play a greater role in sustained engagement.

Current approaches to measuring emotion in digital discourse face several limitations. Sentiment analysis tools trained on Western datasets may not accurately capture emotional expressions in non-Western contexts^28^. Additionally, most studies treat emotion as a static property of content rather than examining how emotional responses evolve as content propagates through networks^29^. The emotion paradox becomes particularly acute in crisis contexts, where preliminary analyses have suggested that neutral content may sometimes generate larger cascade effects than emotionally charged content. This counterintuitive finding challenges dominant theories and highlights the need for more sophisticated approaches to understanding emotion-diffusion dynamics.

### Structural brokers: mediators or agitators?

Structural hole theory, developed by Lazega and Burt^30,31^, positions brokers—individuals who connect otherwise disconnected groups—as having significant social capital due to their unique network positions. These brokers can access diverse information, control information flow between groups, and potentially arbitrage different social worlds. However, the functional roles that brokers actually play, particularly in polarized environments, remain contested. Recent research has identified two primary functions of structural brokers in political networks. Mediating brokers facilitate communication and understanding between different groups, potentially reducing polarization and conflict^32^. In contrast, agitating brokers may exploit their positions to amplify conflict, selectively transmitting inflammatory content to maximize engagement or advance particular agendas^33^.

Empirical studies of broker behavior have yielded mixed results. Shi et al.^34^ analyzed political discussion networks and found that brokers tend to adopt more moderate positions and share more factual information compared to users embedded within homogeneous communities. However, Garimella et al.^35^ found that in highly polarized topics, brokers were more likely to share controversial content and engage in cross-cutting attacks. The behavior of brokers may depend on various factors including the topic’s polarization level, the broker’s own political identity, and the incentive structures of the platform. Research on Twitter during the 2016 U.S. election found that some brokers deliberately amplified divisive content to increase their follower counts and engagement metrics^36^.

The role of brokers in international crisis discourse remains understudied. Preliminary evidence suggests that crisis contexts may create different incentive structures that affect broker behavior. During international conflicts, nationalist sentiments may push brokers toward more agitating roles, while uncertainty and information scarcity may increase the value of mediating brokers who can access and synthesize diverse information sources. Studies of crisis communication have identified the emergence of “information brokers” who specialize in gathering and disseminating crisis-related information^37^. However, these studies have not systematically examined how these information brokers relate to the broader structural broker literature or how their roles may vary across different types of crises.

### Identifying research gaps

Current digital discourse research suffers from three critical limitations: (1) the isolated examination of influence versus homophily, emotion in diffusion, and broker roles, which obscures their crucial interactions—particularly in crisis contexts where emotional brokers may shift between mediating and agitating roles; (2) the predominant focus on Western platforms, limiting generalizability to non-Western digital environments; and (3) the reliance on traditional statistical methods that require a priori specification of variable relationships, potentially missing complex nonlinear interactions between structural, semantic, and emotional factors. Recent advances in Graph Neural Networks, particularly Graph Attention Networks^38^, offer promising solutions by learning rich representations that integrate multiple feature types while preserving relational structure through dynamic relationship weighting^39^, enabling discovery of conditional influence patterns and heterogeneous broker roles that traditional approaches might overlook.

### Research questions

Building on the theoretical foundations and identified gaps, this study addresses three interconnected research questions that integrate the core debates in computational social science:

- RQ1: The Computational Prioritization of Network Signals in Transient Discourse. In a discourse network constructed from transient user interactions, does a Graph Nerual Network trained for link prediction learn to prioritize signals of local homophily over metrics of global prestige (used as a proxy for potential influence)? We investigate this through a dual analysis of the model’s (a) internal attention mechanisms and (b) the decodability of its final learned user representations.
- RQ2: The Affective Nucleus of Homophily. Given that local homophily is the primary organizing principle identified in RQ1, what is its dominant underlying dimension in a polarized conflict discourse? Specifically, by probing the GAT’s learned representations, we test whether affective homophily (connection based on shared emotion) is a more powerful predictor of network ties than structural homophily (connection based on pre-existing community affiliation).
- RQ3: Profiling Emergent Brokers in an Affectively Polarized Network. In a network governed by strong affective homophily, can an analysis of the GAT model’s systematic prediction failures identify a functionally distinct class of users who successfully bridge emotional divides? What are the defining structural, behavioral, and emotional characteristics that distinguish these “emergent anti-homophilic brokers” from traditionally-defined, high-centrality structural brokers?

## Research design

### Data collection and processing

This study analyzes data from Sina Weibo, China’s largest microblogging platform, focusing primarily on discussions related to recent international conflicts. To ensure the domain adaptability of our findings, we also analyze posts from finance and technology domains in the appendix. Data collection was conducted from April 22 to July 1, 2025, using a keyword-based sampling approach. The collected posts centered on international conflicts, specifically focusing on intensely debated topics including the “India-Pakistan conflict,” “Iran-Israel conflict,” and “Russia-Ukraine conflict”. In order to obtain high data quality and reliability of conclusions, we employed a dual-activity sampling strategy. We selected posts with high engagement (reply count > 100), as these typically generate richer user interactions and more diverse opinion exchanges. Additionally, we included low-activity posts (reply count between 5 and 100) to maintain network connectivity, as posts with fewer than 5 replies, while more numerous, would significantly impact network structure by creating overly sparse and disconnected networks. This resulted in a total dataset of 4,176 posts generating 141,732 replies.

To ensure data representativeness and generalizability, we conducted a comprehensive statistical analysis of both posters and repliers. Table 1 presents the demographic and engagement characteristics across different activity levels and post types. This dataset provides a robust foundation for analyzing network formation patterns in highly polarized discourse environments, while the demographic diversity ensures our findings reflect genuine social media dynamics rather than artifacts of specific user populations.

**Table 1:** Characteristics of Posts and Repliers in Social Media Platform.

Activity level	Post type	Posts	Replies	Gender		Prof. cert.		Follower count			
				Male	Female	Yes	No	Ultra-high	High	Medium	Low
High activity	Official media reports	265	27,645	69%	31%	11%	89%	2%	5%	7%	86%
	Opinion leader comments	571	44,661	70%	30%	9%	91%	1%	4%	4%	91%
	Regular user posts	105	18,403	56%	44%	10%	90%	0%	$\sim$0%	1%	98%
Low activity	Official media reports	576	11,239	77%	23%	19%	81%	1%	8%	10%	81%
	Opinion leader comments	1177	19,876	62%	38%	25%	75%	$\sim$0%	5%	7%	88%
	Regular user posts	1482	19,908	59%	41%	8%	92%	0%	0%	1%	99%

Professional certification refers to official domain-specific verification badges granted by the social media platform to users, such as military bloggers, entertainment experts, or social media influencers. Follower count categories are defined as follows: ultra-high (> 500K), High (100K–500K), Medium (10K–100K), and Low (< 10K)

### Network construction

Users were represented as nodes in the interaction network. To capture semantic content, we utilized the bge-large-zh-v1.5^40^ model, which is state-of-the-art, to convert user-generated text into 1024-dimensional embeddings. For users with multiple posts or comments, we employed average pooling across all text embeddings to generate a unified user representation. Each user node was enriched with several key attributes: username identifier, 1024-dimensional text embedding vector capturing semantic content, the proportion of each emotion type, sentiment score, and the traditional structure features.

We constructed a directed network based on two types of user interactions: (1) Comment edges representing “commenter to post author” relationships derived from direct responses to original posts, and (2) Reply edges representing “secondary commenter to primary commenter” relationships derived from nested comment discussions. Edge weights were determined by interaction frequency, with multiple interactions between the same user pair resulting in higher edge weights.

### Sentiment analysis

Sentiment polarity was quantified using two complementary models: Erlangshen-Roberta-110M-Sentiment ^41^ and GISchat-weibo-100k-fine-tuned-bert, both outputting normalized scores $[-1, 1]$. The former leverages robust Erlangshen architecture for Chinese sentiment analysis, while the latter provides domain-specific adaptation through Weibo-based fine-tuning. Fine-grained emotional classification employed the Johnson8187-Chinese-Emotion model, selected for its specialized training on Weibo commentary and capability to distinguish eight emotional states (neutral, concern, happiness, anger, sadness, questioning, surprise, disgust), offering superior granularity over binary classification in capturing social media emotional expressions. A stratified subsample comprising 5% of posts from each emotion class was independently re-annotated by Qwen 3-235B using the prompt in Table S3 of the Supplementary Material. Inter-annotator reliability was high (Cohen’s $\kappa = 0.77$), evidencing substantial concordance between the automated classifier and the supervised labels. The full prompt-engineering workflow is detailed in Table S2.

### Graph attention network model training and evaluation

Model training requires balanced positive and negative samples with a 1:1 ratio. Positive samples consisted of actual edges present in the network, while negative samples were generated using a stratified difficulty-based sampling strategy that incorporates both structural and affective dimensions.

Given a directed interaction graph $G=(V,E)$ with $|E|=m$ observed edges, our goal is to construct a balanced link prediction corpus that: (1) captures realistic structural distances, (2) injects affective signal, and (3) prevents information leakage across data splits. All random operations are executed with seed = 42. For any pair (*u*, *v*), we compute affective similarity and distance using:

1 $$\begin{aligned} \text {sim}(u,v) = \exp \left[ -\text {JS}(\textbf{p}_u,\textbf{p}_v)\right] , \quad \text {dist}(u,v) = -\log \left[ \text {BC}(\textbf{p}_u,\textbf{p}_v)\right] \end{aligned}$$

where $\textbf{p}_u$ and $\textbf{p}_v$ represent the emotional tone probability distributions for nodes *u* and *v* respectively, $\text {JS}$ denotes the Jensen–Shannon divergence measuring distributional dissimilarity, and $\text {BC}$ represents the Bhattacharyya coefficient quantifying distributional overlap.

To establish prediction thresholds, we sample 4,000 random node pairs to derive empirical distributions of similarity and distance metrics, from which we extract three threshold parameters:

2 $$\begin{aligned} T_{\text {med}} = \text {median}(\text {sim}), \quad T_{\text {hard}}^{\text {sim}} = \text {Q}_{25}(\text {sim}), \quad T_{\text {hard}}^{\text {dist}} = \text {Q}_{75}(\text {dist}) \end{aligned}$$

where $T_{\text {med}}$ represents the median similarity threshold, $T_{\text {hard}}^{\text {sim}}$ denotes the 25th percentile similarity cutoff for challenging cases, and $T_{\text {hard}}^{\text {dist}}$ indicates the 75th percentile distance threshold for difficult predictions.

For valence classification, we map the eight fine-grained emotional tones to four coarse valence categories: positive, neutral, negative, and questioning. Node pairs are classified as having *opposite valence* when their dominant emotional tones belong to complementary categories: {(positive, negative), (neutral, negative), (neutral, questioning)}. We generate candidate node pairs for link prediction by identifying all non-adjacent pairs $(u,v) \notin E$ within a shortest path length of 3, computed via breadth-first search traversal. This constraint ensures computational tractability while capturing meaningful proximity relationships in the network structure. The resulting candidate pairs are subsequently stratified into three difficulty categories based on the threshold parameters defined above, as detailed in Table 2.

**Table 2:** Difficulty definition for negative edges. $\text {SPL}$ = directed shortest path length.

Bucket	SPL	Affective filter	Sampling share
Baseline	3	—	0.40*m*
Medium	3	$\text {sim}<T_{\text {med}}$	0.35*m*
Hard	2	$\bigl [\text {sim}<T_{\text {hard}}^{\text {sim}}\bigr ] \vee \bigl [\text {dist}>T_{\text {hard}}^{\text {dist}}\bigr ] \vee \text {opposite valence}$	0.25*m*

If a pool is undersized, we relax $T_{\text {hard}}^{\text {sim}} \leftarrow T_{\text {med}}$ (or $T_{\text {med}} \leftarrow$ 75th-percentile) once; remaining deficits are accepted and reported. $\langle \text {train}:\text {val}:\text {test}\rangle = \langle 0.70:0.15:0.15\rangle$. Positive edges inherit their endpoints’ split, whereas each negative bucket is re-shuffled to respect the same ratio. This *node-wise* split guarantees that test nodes are completely unseen during training. We employed the several Graph Neural Networks architecture with two convolutional layers and implemented a three-stage progressive training strategy. We implement an adaptive curriculum learning framework that dynamically adjusts the training difficulty based on model performance progression. The training process employs a gradual difficulty escalation mechanism with the following key components The training begins with exclusively easy negative samples (hard ratio $r_h = 0.0$) and progressively incorporates more challenging examples. At every $\tau = 5$ epochs, the system evaluates validation performance and applies the following adaptation rule:

3 $$\begin{aligned} r_h^{(t+1)} = {\left\{ \begin{array}{ll} \min (r_h^{(t)} + \Delta r, 0.5) & \text {if } \text {AUC}^{(t)} < \text {AUC}^{(t-\tau )} - \epsilon \\ r_h^{(t)} & \text {otherwise} \end{array}\right. } \end{aligned}$$

where $r_h^{(t)}$ denotes the hard sample ratio at stage *t*, $\Delta r = 0.1$ represents the difficulty increment, $\epsilon = 0.003$ is the performance threshold, and the maximum hard ratio is capped at $50\%$ to maintain training stability. At each training stage, the negative sample distribution follows a structured allocation strategy. Given a hard ratio $r_h$, the sample composition is determined as:

4 $$\begin{aligned} {\left\{ \begin{array}{ll} n_{\text {hard}} = \min (\lfloor N \cdot r_h \rfloor , |S_{\text {hard}}|) \\ n_{\text {medium}} = \min (\lfloor N \cdot 0.3 \rfloor , |S_{\text {medium}}|) \\ n_{\text {easy}} = N - n_{\text {hard}} - n_{\text {medium}} \end{array}\right. } \end{aligned}$$

where *N* represents the total number of positive samples (maintaining class balance), and $|S_{\text {difficulty}}|$ denotes the available samples in each difficulty stratum. The framework incorporates several stabilization techniques: (1) early stopping with patience parameter $p = 20$ epochs to prevent overfitting, (2) maximum training duration of $T_{\max } = 200$ epochs, and (3) best model checkpointing based on validation AUC performance. The optimizer employs AdamW with learning rate $\eta = 5 \times 10^{-4}$ and weight decay $\lambda = 10^{-4}$ for regularization.

To identify the optimal graph neural network architecture for link prediction, we conducted a comprehensive comparative analysis across multiple model paradigms. Our evaluation framework encompasses both traditional machine learning baselines and state-of-the-art graph neural network architectures. Specifically, we employed logistic regression and random forest as baseline methods to establish performance benchmarks, while evaluating four distinct GNN variants: Graph Convolutional Networks (GCN)^42^, GraphSAGE^39^, Graph Attention Networks v2 (GATv2)^43^, and GATv2 with GraphSAINT sampling (GATv2-SAINT)^44^. The experimental design systematically assessed model performance across three difficulty levels—baseline, medium, and hard—using four evaluation metrics: F1-score, recall, accuracy, and precision. The experimental results in Table 3 demonstrate that traditional machine learning baselines exhibit significant performance degradation with increasing task difficulty, with logistic regression declining from 0.82 to 0.58 F1-score. Among GNN architectures, GATv2 achieves superior and consistent performance across all difficulty levels (F1-scores: 0.87, 0.82, 0.85), substantially outperforming both baselines and other GNN variants on challenging scenarios. We chose GATv2 not only for its outstanding predictive performance, but also because it offers an interpretable diagnostic framework. Unlike traditional methods, GNNs allow us to explore their internal decision-making mechanisms through attention analysis and embedding decoding. It is precisely this diagnostic process that enables us to clearly observe that the GAT model captures the network connection patterns by prioritizing the encoding of emotional similarity between nodes, thereby explaining the phenomenon of ’emotion outweighing reputation’ that was not reflected in traditional methods.

**Table 3:** Performance comparison of GNN models across different difficulty levels.

	Baseline				Medium				Hard			
Model	F1	Recall	Acc.	Prec.	F1	Recall	Acc.	Prec.	F1	Recall	Acc.	Prec.
Logistic regression	0.82	0.81	0.85	0.83	0.75	0.73	0.78	0.77	0.58	0.56	0.61	0.60
Random forest	0.88	0.86	0.80	0.80	0.71	0.79	0.73	0.73	0.64	0.62	0.66	0.66
GCN	0.81	0.89	0.83	0.83	0.82	0.83	0.87	0.84	0.72	0.71	0.71	0.71
GraphSAGE	0.83	0.82	0.85	0.84	0.81	0.82	0.80	0.80	0.80	0.79	0.79	0.82
GATv2	0.87	0.86	0.89	0.88	0.82	0.85	0.85	0.87	0.85	0.83	0.85	0.82
GATv2-SAINT	0.85	0.84	0.87	0.86	0.83	0.81	0.85	0.85	0.78	0.79	0.81	0.82

## Results

In a real-world online conflict network, traditional network theories are becoming inadequate. We trained a Graph Neural Network (GNN) to model the fundamental process of “edge formation,” functioning as a “computational sociologist” to uncover the true forces driving network interactions. This section presents the core empirical findings of this study.

### RQ1: the computational primacy of homophily over structural prestige

To investigate how Graph Attention Networks (GATs) prioritize different network signals during link prediction, we analyzed the attention mechanisms across two layers of our trained GATv2 model. Following the interpretability framework proposed by Agarwal et al.^45^, we examined the alignment between the model’s explicit explanations (attention weights) and various network properties using Spearman rank correlation analysis. This approach provides direct insight into the model’s decision-making process—rather than inferring feature importance from outputs, we observe which neighbor characteristics the model actively attends to during message aggregation. It should be emphasized that our analysis reveals computational associations, not causal mechanisms. The correlations between attention weights and homophily indicators demonstrate what patterns the GNN learns to exploit for prediction accuracy, not why these connections form in the real network. The behavior of this model reflects the statistical patterns in the data. These patterns, under the constraints of the graph attention framework, are proven to be practical for link prediction in multiple experiments^46,47^.

We first established the baseline community structure using the Infomap algorithm^48^, which identified 109 distinct communities with a modularity score of $Q = 0.79$, indicating well-defined community boundaries with strong intra-community connectivity and sparse inter-community links. After normalizing node features, we decoded the attention weights $\alpha _{ij}$ from both GAT layers, where $\alpha _{ij}$ represents the importance assigned by node *i* to its neighbor *j* during message aggregation. For each edge (*i*, *j*) in the graph, we computed Spearman’s rank correlation coefficient $\rho$ between the attention weights and the following node-pair characteristics: Community co-membership, sentiment similarity (both binary and continuous), and dominant emotion alignment; Target node’s PageRank, HITS authority/hub scores, clustering coefficient, and post count. The choice of Spearman’s $\rho$ over Pearson’s correlation is motivated by its robustness to non-linear relationships and outliers, making it particularly suitable for analyzing attention distributions which often exhibit heavy-tailed behavior.

**Table 4:** Spearman rank correlations ($\rho$) of selected features with attention weights in two GAT layers.

Metric	Layer 1 $\rho$	Layer 1 *p*-value	Layer 2 $\rho$	Layer 2 *p*-value
Community homophily	0.298	0.004**	0.626	$<0.001$***
Sentiment homophily (binary)	0.286	0.011*	0.706	0.046*
Sentiment similarity (continuous)	0.289	$<0.001$***	0.521	$<0.001$***
Dominant-emotion homophily	0.237	$<0.001$***	0.544	$<0.001$***
Target PageRank	− 0.092	$<0.001$***	− 0.089	$<0.001$***
Target authority	0.088	0.048*	− 0.185	$<0.001$***
Target hub score	0.056	0.007	0.269	$<0.001$***
Target clustering coefficient	− 0.031	$<0.001$***	0.208	0.071
Target post count	− 0.069	$<0.001$***	− 0.283	0.301

Statistical significance: ***$p<0.001$, **$p<0.01$, *$p<0.05$. All tests are two-tailed with Bonferroni correction. Homophily features measure similarity between node pairs: community co-membership (binary), sentiment alignment (binary: same polarity; continuous: $1-|\Delta _{sentiment}|$), and dominant emotion match (argmax over 8 emotion categories). Structural features capture target node’s network position: PageRank (global importance), HITS authority/hub scores (information source/aggregator roles), clustering coefficient (local density), and post count (activity level). Attention weights $\alpha _{ij}$ represent averaged multi-head attention across 4 heads per layer.

The results in Table 4 reveal a striking pattern: the GAT’s attention mechanism exhibits strong positive correlations with homophily-based features while showing negligible or negative correlations with structural prestige indicators. Specifically: Homophily signals become increasingly dominant from Layer 1 to Layer 2. Community co-membership correlation increases from $\rho = 0.298$ to $\rho = 0.626$, suggesting that deeper layers learn to prioritize local similarity over global structure. PageRank, the quintessential measure of global importance, maintains weak negative correlations across both layers ($\rho \approx -0.09$), indicating that the model actively discounts information from high-prestige nodes when making predictions. Binary sentiment homophily shows the highest correlation in Layer 2 ($\rho = 0.706$), suggesting that emotional alignment between nodes serves as a powerful signal for link formation in this network.

These findings provide direct evidence of the model’s internal decision-making process. Rather than inferring behavior from final embeddings, we observe the GAT’s reasoning through its attention allocation strategy. The model has discovered that, for the task of link prediction in this social network, homophily provides actionable signals while structural prestige offers little predictive value. This computational behavior aligns with recent theoretical work suggesting that GNNs implicitly perform a form of spectral clustering that naturally emphasizes local neighborhood similarity^49^. The implications are profound: when tasked with learning representations for generative purposes, GNNs may systematically privilege local homophilic patterns over global structural properties, potentially missing important hierarchical or influence-based dynamics in the network. This selective attention mechanism represents not a limitation but rather a rational adaptation to the objective function—maximizing link prediction accuracy by focusing on the most informative signals available.

### RQ2: the affective nucleus of community structure

Having established the computational primacy of homophily over structural prestige, we further investigated which dimensions of homophily dominate the learned representations. To probe the information encoded in GAT embeddings, we employed linear decodability analysis—training simple linear classifiers to predict node attributes from the learned representations. This approach reveals what information the model prioritized during its link prediction training.

The results presented in Table 5 demonstrate a striking asymmetry between emotion and community decodability. The 8-category emotion classification achieved an F1 score of 0.571 (±0.011), with precision of 0.631 and recall of 0.562, indicating that fine-grained emotional states are robustly preserved in the embeddings. In contrast, community membership prediction performed significantly worse, with macro-averaged F1 of only 0.193 (±0.010) and micro-averaged F1 of 0.227 (±0.003). This represents a 247.5% F1 improvement in emotion decodability relative to community structure, providing compelling evidence that GAT’s attention mechanism prioritizes node-level semantic attributes over network topology. Notably, this disparity persists despite the greater complexity of 8-class emotion classification compared to binary sentiment analysis, suggesting that GAT embeddings capture nuanced emotional distinctions rather than merely sentiment polarity.

**Figure 1:**
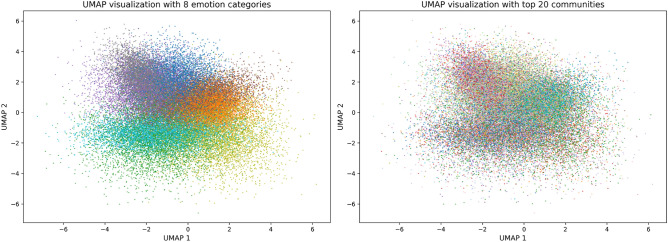
UMAP visualization of GAT embeddings colored by (**a**) dominant emotion categories and (**b**) top 20 community assignments. The emotion-based visualization reveals clear clustering patterns, while community boundaries appear dispersed throughout the embedding space.

**Table 5:** Linear decodability performance of GAT embeddings across emotion and community classification tasks.

Task	Type	Precision	Recall	F1-Score
Emotion	8 Categories	0.631	0.562	0.571 ± 0.011
Community	Macro avg	0.177	0.198	0.193 ± 0.010
Community	Micro avg	0.254	0.252	0.227 ± 0.003

*Note:* Results averaged over 10-fold cross-validation. Emotion categories include neutral, concerned, happy, angry, sad, questioning, surprised, and disgusted. Community classification involves 109 detected communities

The UMAP visualizations in Fig. 1 provide further confirmation of emotion-centric organization. When colored by dominant emotion (left panel), the embedding space exhibits clear clustering with smooth transitions between emotional states, suggesting that the GAT has learned a coherent emotional topology. Conversely, when colored by community membership (right panel), the same embedding space shows a scattered distribution with no discernible community-based clustering. This visual evidence corroborates our quantitative findings: the GAT model, optimized for link prediction, discovered that encoding users’ emotional states provides more actionable information than encoding their community affiliations. These findings offer computational support for reconceptualizing online echo chambers as “emotional fortresses”—spaces where shared affect, rather than traditional group boundaries, drives network formation and information flow.

### RQ3: the discovery of emergent anti-homophilic brokers

While GAT models excel at learning dominant network formation mechanisms, their systematic prediction failures reveal alternative pathways of connection that defy prevailing patterns. By analyzing edges that the model confidently predicted as unlikely (probability< 0.205, 10th percentile), we discovered a distinct class of “emergent brokers” who successfully bridge emotional divides despite lacking traditional structural advantages. These 1,139 users, identified through their participation in 2998 “surprise edges,” represent a fundamentally different brokerage mechanism from the 5,831 traditional brokers identified via high betweenness centrality.

The comparative analysis presented in Table 6 reveals striking differences between these two broker types. Emergent brokers exhibit systematically lower structural centrality across all measures: their betweenness centrality is 66.7% lower than traditional brokers, degree centrality is 70.0% lower, and PageRank authority is 80.9% lower. These differences are not merely statistical artifacts but represent a fundamental distinction in how these actors position themselves within the network. Traditional brokers occupy structurally advantageous positions at the intersection of multiple communities, leveraging their high connectivity to bridge different network regions. In contrast, emergent brokers operate from the periphery, maintaining sparse connections that nonetheless prove crucial for cross-group communication.

**Table 6:** Structural and behavioral characteristics of emergent vs. traditional information brokers.

Network metric	Emergent brokers	Traditional brokers	Effect size	Significance
Structural centrality measures				
Betweenness centrality	0.0001 ± 0.0002	0.0003 ± 0.0004	− 66.7%	p< 0.001***
Degree centrality	0.003 ± 0.002	0.010 ± 0.008	− 70.0%	p< 0.001***
PageRank authority	6.98e−06 ± 1.2e−05	3.66e−05 ± 2.1e−05	− 80.9%	p< 0.001***
HITS authority	1.15e−11 ± 2.3e−11	4.39e−11 ± 3.8e−11	− 73.8%	p< 0.001***
Behavioral activity measures				
Total posts	2.0 ± 1.8	1.0 ± 1.2	+100.0%	p = 0.580
Sentiment volatility	0.438 ± 0.085	0.458 ± 0.092	− 4.4%	p< 0.05*

Emergent brokers (n = 1139) identified through GAT model prediction failures on emotionally polarized connections (predicted probability< 10th percentile). Traditional brokers (n = 5831) selected based on high betweenness centrality scores. Values shown as median ± IQR. Mann-Whitney U tests performed for all comparisons. Statistical significance: ***p < 0.001, *p < 0.05.

**Figure 2:**
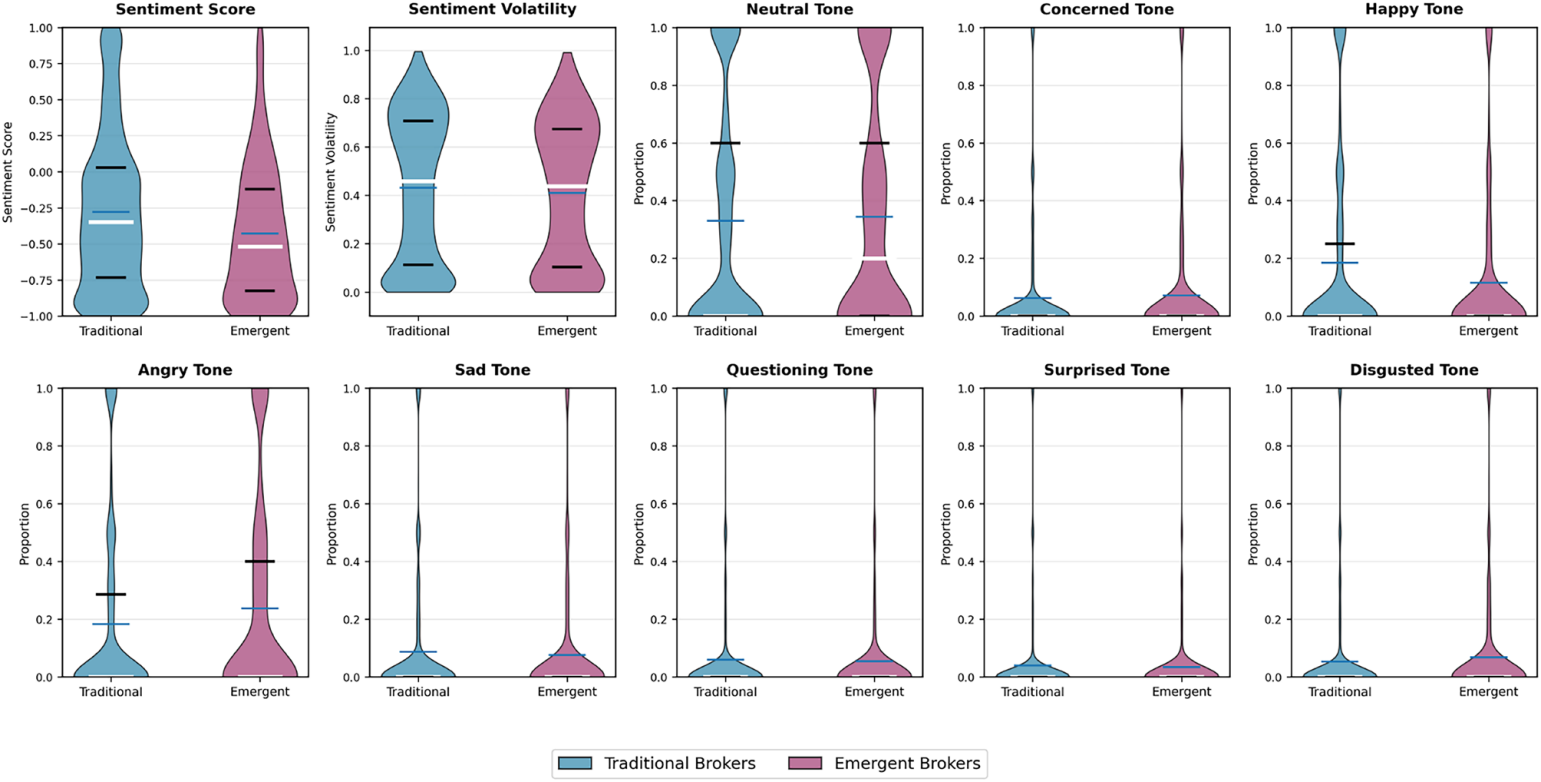
Emotional profiles of emergent vs. traditional brokers across multiple dimensions. Emergent brokers exhibit more neutral sentiment positioning and lower emotional intensity, enabling them to bridge emotionally polarized communities despite lacking structural advantages.

The emotional profiles shown in Fig. 2 provide crucial insights into the mechanisms enabling emergent brokers to transcend homophilic barriers. While both broker types exhibit similar sentiment volatility (0.438 vs 0.458, p< 0.05), their emotional positioning differs substantially. Emergent brokers maintain more neutral sentiment scores (median closer to zero) and demonstrate higher proportions of neutral emotional tone. This emotional neutrality serves as their primary asset—by avoiding strong emotional signals that would align them with specific groups, they remain accessible to users across the emotional spectrum.

The existence of these emergent brokers challenges conventional network theory in several ways. First, they demonstrate that effective brokerage does not require structural privilege—peripheral actors can facilitate crucial cross-group connections through behavioral strategies rather than positional advantages^30^. Second, their success reveals the limitations of homophily-focused models: while the GAT correctly learned that most connections form between emotionally similar users, it systematically underestimated the connective potential of emotionally neutral actors^50^. Third, these findings suggest that in highly polarized networks, “invisible bridges” maintained by low-profile, emotionally moderate users may be as important for network cohesion as the visible connections maintained by structural elites^51^. This discovery has profound implications for understanding information flow in polarized online environments. While traditional brokers may efficiently transmit information within their extensive networks, emergent brokers provide the weak ties that prevent complete network fragmentation. Their strategy of emotional neutrality and behavioral moderation allows them to circumvent the “affective barriers” that increasingly segment online discourse. In essence, they represent a form of social capital that operates not through accumulation of connections or influence, but through strategic positioning at the interstices of emotional divides—serving as the “diplomatic corps” of polarized digital spaces^52^.

## Discussion

### The contingency of network principles: boundary conditions of the emotional fortress

Our findings reveal that in high-polarization crisis contexts, social networks crystallize into “emotional fortresses”—homogeneous clusters where affect-based homophily dominates traditional structural principles of network formation. This affect-first dynamic challenges established network theories that prioritize structural advantages like brokerage positions^30^ and prestige-based attachment^18^. The dominance of emotional similarity in predicting link formation aligns with research on emotional contagion in social media^20,23^, but extends these findings by demonstrating how emotions fundamentally restructure network topology during polarized crises. Notably, our identification of affect-neutral brokers—peripherally positioned actors who leverage emotional neutrality rather than structural centrality—suggests that traditional concepts of network advantage^51,52^ may require reconceptualization when emotional polarization renders structural positions ineffective.

However, these affect-first dynamics represent a contingent rather than universal principle, as our boundary analysis (Table 7) and supplementary experiments demonstrate. In information-driven contexts like finance and technology discussions (Table S1 of Supplementray Material), structural prestige metrics overwhelmingly predict network attention allocation, confirming that expertise and authority remain primary connective forces when emotional stakes are lower. This contingency extends across multiple dimensions: platform affordances that amplify emotional visibility^21^, analytic tasks that reveal different network mechanisms, and broker types that operate through distinct logics. The theoretical implication is profound—network formation principles are not fixed but dynamically shift based on issue polarization, platform design, and social context. Future research should explore how these boundary conditions interact, particularly examining whether the emotional fortress phenomenon manifests differently across cultural contexts^4,27^ and how platform-specific features might amplify or attenuate affect-driven network segregation.

**Table 7:** Boundary conditions of the “emotional fortress” model.

Dimension	Condition	Dominant principle	Theoretical rationale	Evidence
Issue context	High-polarization crisis	Affect-first	Emotional arousal depletes cognitive resources; users rely on affective shortcuts	Current study
	Information-driven topics	Structure-first	Expertise and authority drive connections when emotions are low	Appendix A
Platform design	Public squares (Weibo, Twitter)	Affect-first	Trending algorithms and viral mechanics amplify emotional content	Current data
	Professional networks (LinkedIn)	Structure-first	Identity verification and expertise signals favor prestige	Cross-platform study*
Network task	Link prediction	Affect-first	Emotional similarity predicts new connections in polarized contexts	RQ1-RQ2
	Cascade dynamics	Mixed	Authority initiates; emotion constrains paths	Future work*
Broker type	Structural brokers	Position-based	High betweenness actors bridge via network centrality	Traditional theory
	Affect-neutral brokers	Emotion-based	Peripheral actors bridge via emotional neutrality	RQ3 (novel finding)

*Indicates areas for future research. The table synthesizes how network formation principles shift from structure-dominant to affect-dominant based on contextual factors.

### Limitations

Firstly, although the data source of this study is extensive in volume, it is still limited to a single platform (Sina Weibo) and a specific time period (from April to July 2025) and a specific topic (international conflicts). This restricts the direct generalization ability of the research conclusions, as we discussed in the previous section. Secondly, although GNN provides us with a powerful analytical tool to observe the “computational decision-making” process of the model, we must be cautious and cannot equate the “attention” of the model with the “cognitive intention” of humans. The correlations discovered by the model are statistical patterns in the data, rather than strict causal mechanisms. Finally, our analysis of the “emergent brokers” focuses on their network structure and quantified emotional expressions. However, the motivations and specific communication strategies behind why they choose and how they maintain this emotionally neutral stance still need to be further explored through qualitative research methods such as in-depth interviews.

### Future research

Building on our findings, we outline several promising avenues for future research to deepen understanding of affect-driven network formation and its boundary conditions.(1) Systematic validation of the emotional fortress model requires examination across diverse contexts. Future research should apply our framework to varied domains (finance, technology, health) and platform types (professional networks vs. public forums) to map how emotional versus structural factors shift across contexts. Such comparative work would establish a typology of network formation regimes, moving beyond binary affect-versus-structure distinctions. (2) Moving from correlation to causation, future work should employ experimental designs. Online field experiments could test whether algorithmic exposure to emotionally neutral content from opposing viewpoints facilitates “surprise edge” formation. Natural experiments leveraging platform policy changes could provide additional causal evidence while informing practical interventions for reducing polarization. (3) Our static snapshot misses crucial temporal dynamics. Future research should construct temporal networks to track how emotional fortresses emerge, solidify, and potentially dissolve. Key questions include: How quickly do these structures form? Is affect-neutral brokerage a stable position? Stochastic actor-oriented models^16^ or temporal GNNs could reveal the co-evolution of emotions and network structure.

## Conclusion

This study demonstrates how graph neural networks can become diagnostic frameworks for uncovering social mechanisms, revealing the emergence of “emotional fortresses” during international conflicts where affect-based homophily overwhelms traditional structural principles of network formation. Our discovery of affect-neutral brokers—peripheral actors who bridge polarized communities through emotional positioning rather than structural advantage—challenges conventional network theories and suggests new pathways for reducing polarization. Through cross-domain analysis, we establish that network organizing principles are fundamentally context-dependent: structure dominates in knowledge-sharing environments while affect dominates in crisis contexts. These findings advance both computational social science methodology and our theoretical understanding of how emotions reshape digital networks during polarized global events.

## Supplementary Information

Supplementary Information.

## Data Availability

The datasets generated and analyzed during the current study are not publicly available due to privacy considerations related to social media user data, but are available from the corresponding author on reasonable request, subject to appropriate data sharing agreements and ethics approvals. The authors are committed to sharing these datasets with qualified researchers for legitimate research purposes. Preliminary code for data collection and Graph Attention Network implementation is available at: https://github.com/ZachGu-00/weibo-sentiment-gat. Please note that the current repository is under active development and contains initial implementations adapted for Chinese social media platforms. The repository currently lacks complete analysis code components, which will be updated and made available following manuscript acceptance. The code is provided to support reproducibility and facilitate future research in computational social science.
