# Supplementary material for: Affective homophily as the dominant organizing principle in online conflict discourse networks
Source: Sci Rep. 2025 Oct 10;15:35542. doi: 10.1038/s41598-025-19605-3 (PMC12514174; doi:10.1038/s41598-025-19605-3)
Supplement: Supplementary file 1 — Supplementary Information. [file 41598_2025_19605_MOESM1_ESM.pdf]

Supplementary Material: Affective Homophily as the Dominant Organizing Principle in Online Conflict Discourse Networks

Table S1: Spearman rank correlations (ρ) of selected features with GAT attention weights: Finance vs. Technology domains

**Metric**	**Finance**			**Technology**		
	**Layer 1** ρ	**Layer 2** ρ	p**-value**	**Layer 1** ρ	**Layer 2** ρ	p**-value**
Community Homophily	0.164	0.287	0.012*	0.142	0.251	0.024*
Sentiment Homophily (binary)	0.098	0.189	0.086	0.076	0.134	0.167
Sentiment Similarity (continuous)	0.127	0.203	0.041*	0.089	0.156	0.098
Dominant-Emotion Homophily	0.082	0.145	0.134	0.063	0.108	0.245
Target PageRank	0.642	0.656	< 0.001***	0.889	0.824	< 0.001***
Target Authority	0.698	0.687	< 0.001***	0.712	0.721	< 0.001***
Target Hub Score	0.545	0.534	< 0.001***	0.667	0.598	< 0.001***
Target Clustering Coefficient	0.198	0.289	0.003**	0.224	0.315	0.001**
Target Post Count	0.156	0.201	0.047*	0.143	0.187	0.072
**GATv2 Model Accuracy**		**0.74**			**0.72**	

Note. Statistical significance: ***$p < 0.001$, **$p < 0.01$, *$p < 0.05$.

Table S2: Confusion matrix: Automated vs. LLM-supervised emotion classification

**Automated\LLM**	**Neu**	**Con**	**Hap**	**Ang**	**Sad**	**Que**	**Sur**	**Dis**	**Total**
Neutral	**1068**	87	8	12	23	7	21	19	1245
Concerned	125	**754**	25	53	22	20	5	0	979
Happy	1	27	**685**	33	24	5	49	0	824
Angry	84	11	3	**803**	77	0	0	0	978
Sad	87	32	0	74	**655**	2	0	0	850
Questioning	92	17	22	11	9	**613**	17	15	796
Surprised	33	14	11	15	29	54	**600**	23	779
Disgusted	76	0	0	0	62	8	0	**489**	635
**Total**	1104	1041	860	942	880	808	783	668	**7086**

Note. Validation on a stratified random sample of 7086 posts (5% of dataset). Bold diagonal = correct classifications. Cohen's $\kappa = 0.77$.

Table S3: Prompt template for emotion classification validation with Qwen3-235B

Part	Detail
**System Role**	You are a senior Chinese sentimentanalysis expert studying affective patterns in socialmedia language. Classify each short Weibo comment into one of eight emotion categories.
**Emotion Categories**	1. **Neutral tone** – objective statement with no clear emotion<br>2. **Concerned tone** – expressing anxiety, worry or concern<br>3. **Happy tone** – positive, conveying joy or satisfaction<br>4. **Angry tone** – dissatisfaction, indignation or strong opposition<br>5. **Sad tone** – disappointment, depression or sorrow<br>6. **Questioning tone** – doubt, query or request for clarification<br>7. **Surprised tone** – shock, unexpectedness or amazement<br>8. **Disgusted tone** – dislike, contempt or strong aversion
**Guidelines**	• Analyse the overall sentiment of the text<br>• Pay attention to emojis, punctuation and internet slang<br>• Consider context and implied meaning<br>• Choose the single most prominent emotion
**Response Format**	**Classification**: [one of the 8 categories]<br>**Key rationale**: [brief justification, 50 characters max]
